# A New Approach to Assess Blinding for Transcranial Direct Current Stimulation Treatment in Patients with Fibromyalgia. A Randomized Clinical Trial

**DOI:** 10.3390/brainsci11101335

**Published:** 2021-10-11

**Authors:** Rubén Arroyo-Fernández, Juan Avendaño-Coy, Rafael Velasco-Velasco, Rocío Palomo-Carrión, Elisabeth Bravo-Esteban, Asunción Ferri-Morales

**Affiliations:** 1 Physiotherapy Unit, Nª Sª del Prado Hospital, 45600 Talavera de la Reina, Spain; Ruben.Arroyo@uclm.es; 2 Faculty of Physiotherapy and Nursing, University of Castilla-La Mancha, 45071 Toledo, Spain; Rafael.Velasco@uclm.es (R.V.-V.); Rocio.Palomo@uclm.es (R.P.-C.); Elisabeth.Bravo@uclm.es (E.B.-E.); Asuncion.Ferri@uclm.es (A.F.-M.); 3 Toledo Physiotherapy Research Group (GIFTO), 45071 Toledo, Spain; 4 Health and Social Research Center, University of Castilla-La Mancha, Camino de Pozuelo, 16071 Cuenca, Spain

**Keywords:** transcranial direct current stimulation, fibromyalgia, placebo, blinding, James’ index, Bang’s index

## Abstract

Correct blinding is essential for preventing potential biases. The aim of this study was to assess the blinding of participants and a therapist following treatment with transcranial direct current stimulation in subjects with fibromyalgia using James’ and Bang’s blinding indexes. Eighty subjects were randomly allocated either active or sham stimulation groups in an intervention of five sessions lasting 20 min each. A questionnaire was delivered to both the therapist and patients after the last session to record their guess of which treatment had been applied. No differences between the groups were noted at baseline in terms of demographic or clinical data. James’ BI was 0.83 (CI 95%: 0.76–0.90) for the patients and 0.55 (CI 95%: 0.45–0.64) for the therapist. Bang’s BI for subjects was −0.08 (CI 95%: −0.24–0.09) and −0.8 (CI 95%: −0.26–0.1) for the active and sham transcranial direct current stimulation groups, respectively. Bang’s BI for the therapist was 0.21 (CI 95%: −0.02–0.43) and 0.13 (CI 95%: −0.09–0.35) for the active and sham transcranial direct current stimulation groups, respectively. Protocols of active and sham transcranial direct current stimulation applied in this study have shown satisfactory blinding of the therapist and subjects with fibromyalgia.

## 1. Introduction

Transcranial direct current stimulation (tDCS) is a non-invasive brain stimulation technique used in humans since the beginning of the twenty-first century. It consists of low-intensity galvanic currents applied on the cranium via a set of electrodes [1,2], which modulates the excitability of the brain cortex underneath them [2]. Its effectiveness, safety, and easy application make it a key therapy [3,4,5] in the treatment of chronic pain, whose effects on fibromyalgia have been assessed by several randomized clinical trials (RCTs) [6,7,8,9,10].

Ensuring correct blinding that conceals the assigned group to patients and researchers is essential for preventing biases in clinical trials [11,12]. Blinding is less often reported in RCTs assessing nonpharmacological treatments, possibly due to the difficulty in design or the lack of knowledge [13,14]. Creating a robust blinding method is one of the main demands for researchers to validate tDCS as an effective treatment in phase III of clinical trials [5]. To achieve this, research protocols must include an assessment of the blinding methods using statistical models that determine their reliability and effectiveness.

James’ and Bang’s blinding indexes (BIs) are the two statistical methods most widely employed for quantifying the effectiveness of blinding in clinical trials [15], employing subjective ad hoc data of subjects and researchers [16,17]. Up until now, no RCT on tDCS has utilized these methods to conduct a statistical analysis of the blinding of subjects or researchers.

The aim of this trial was to assess the blinding of both the subjects and therapist in the tDCS treatment of patients with fibromyalgia.

## 2. Methods

### 2.1. Study Design

The study was designed as a randomized, triple-blind (subject, therapist, evaluator), sham-controlled clinical trial. The study protocol was approved by the Ethics Committee for clinical trials of the health area Talavera de la Reina in Spain (registration number 13/2019). All the procedures were conducted according to the Declaration of Helsinki. This study was also registered in the Cliniclatrials.gov database (NCT04050254). The reporting was conducted in accordance with Consolidated Standards of Reporting Trials (CONSORT) and the recommendations for randomized trials.

### 2.2. Participants and Settings

The study subjects were volunteers, treatment-naive *for tDCS* and diagnosed with fibromyalgia, who complied with the inclusion criteria and provided written consent prior to their recruitment for the trial.

The criteria for the inclusion of participants were: (1) age between 18 and 65 years; (2) diagnosed with fibromyalgia following the criteria by the *American College of Rheumatology* [18]; (3) reported usual pain intensity of ≥4 points on the visual analog scale; (4) capable of participating in a therapeutic exercising program; (5) properly understood spoken and written Spanish. Criteria for exclusion were: pregnancy or breastfeeding; metal implants in the head; moderate-to-severe brain trauma or brain surgery; brain tumor, epilepsy, or stroke; a history of drug use in the last six months; carbamazepine consumption in the last six months; severe depression (Beck depression inventory II ≥ 29); diagnosed with psychiatric disorder; non-controlled rheumatologic pathology; coexisting autoimmune pathology or chronic inflammatory disease (rheumatoid arthritis, lupus, inflammatory bowel disease).

Recruitment and inclusion in the study commenced in September of 2019 following informative sessions that took place in fibromyalgia patients associations. The intervention and evaluations were conducted in one primary healthcare center up to March of 2020. Participants were considered as losses to follow-up when they missed the treatment two or more days [9].

### 2.3. Sample Size Calculation

Sample size was calculated with reference to a previous study conducted by Larsson et al. [19] in which pain was measured in fibromyalgia patients using a visual analog scale in millimeters for general current pain. In this study, a mean difference of 14.8 mm was recorded, with standard deviations of 25.2 mm in the intervention group and 20.0 mm in the control group. Furthermore, a type I error of 5% and a type II error of 20% were set. This calculation rendered 37 participants per group. Ultimately, three participants were included in each group to prevent loss of power derived from potential dropouts. Epidat version 4.0 was used for sample size estimation (Xunta de Galicia, Servizo Galego de Saúde, Spain).

### 2.4. Randomization and Blinding

Subjects were randomly allocated into one of three intervention groups (active tDCS, sham stimulation, and control), although the evaluation of blinding effectiveness was exclusively conducted for the preliminary outcomes of the active and sham tDCS groups since subjects assigned to the control group were aware of their assignment. One researcher (J.A.-C.) employed software Epidat 4.0 for the correct allocation of subjects into each group so that the number of participants was equal in all groups and concealed from the research team. Another researcher, R.V.-V., applied the interventions, while the assessment of the outcome variables was conducted by R.A.-F. The statistical analysis of the outcomes was also performed by J.A.-C., the researcher who was not blind to the allocation.

To ensure the blinding of the therapist and subjects, an external researcher previously programmed the ‘double-blind mode’ available in the software interface of the device, which allows for the automatic generation of active or sham stimulation after adjusting the parameters for both options. The system can be run in a special password-protected mode that minimizes the information presented on the screen.

### 2.5. Intervention

**Figure 1:**
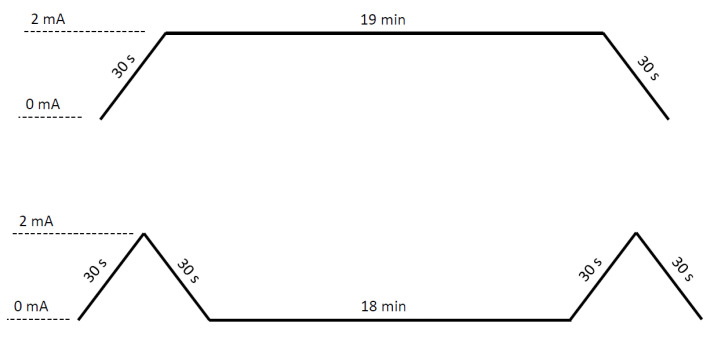
Stimulation protocol. Active tDCS (upper) and sham tDCS (lower).

The intervention lasted two weeks and comprised five sessions of tDCS stimulation (active or sham), each lasting 20 min, three in the first week and two in the second week, on alternate days. A STARSTIM^®^ 8 (Neuroelectrics, Barcelona, Spain) stimulator was used to apply the tDCS. The device was programmed following the FISSFO protocol [20]. To apply active tDCS, the current intensity was ramped up for 30 s to reach 2 mA, which was maintained until the end of the session, when a 30-s ramp-down period was set to reach 0 mA. The sham tDCS group used a 30-s ramp-up to reach 2 mA current intensity followed immediately by a 30-s ramp-down to 0 mA; this ramp-up and ramp-down activity was performed at both the beginning and end of the session (Figure 1). Circular sponge electrodes of 25 cm^2^ (SPONSTIM, Neuroelectrics, Barcelona, Spain) were applied in both groups through the headcap the device provides. The anode was placed at the level of M1 (primary motor cortex) of the left hemisphere (C3 position according to the international 10–20 system for placing scalp electrodes), and the cathode was placed at the level of the right supraorbital area (Fp2 position) [9,10,21,22,23,24]. Prior to current delivery, electrodes were soaked in 15 mL of sterile sodium chloride solution (0.9%).

### 2.6. Outcome Variables

Demographic and clinical data recorded at baseline were age, gender, body mass index, time since being diagnosed with fibromyalgia, Widespread Pain Index, Symptoms Severity Scale, pain intensity (measured on a Visual Analogue Scale), quality of life (measured via the Fibromyalgia Impact Questionnaire), anxiety (as per the State–Trait Anxiety Inventory), pain catastrophism (measured via the Pain Catastrophizing Scale), and depression (measured according to the Beck Depression Inventory II).

Both the participant and therapist were questioned separately after the last session to evaluate the blinding success. Following the recommendations by Bang et al. [25] and Kolahi et al. [26], a close-ended questionnaire was devised to enquire about their treatment assignment. First, they were asked, ‘What treatment do you believe you have received?’, where they could choose from three possible answers: (1) real current; (2) simulated current; (3) do not know. When the participants or therapist answered, ‘*Do not know*’, they were re-asked about their treatment guess one more time with the question ‘If your answer was ‘*Do not know*’, would you be willing to provide your best guess about the treatment you received/applied?’, and could choose from two possible replies: (1) real current; (2) simulated current.

The researcher who carried out the evaluations remained blinded with respect to the group allocation. The therapist who applied the intervention, therefore, did not carry out any of the evaluations.

### 2.7. Statistical Analysis

A descriptive analysis of demographic and clinical variables and a comparison between groups (active and sham) was performed at baseline. Student’s *t*-test and Chi-squared test were employed in the case of quantitative and qualitative variables, respectively.

To analyze the blinding outcome variable, James’ BI [16] and Bang’s BI [17,25] were obtained using Stata v15.0 (StataCorp, Texas, USA). James’ BI is used to infer the overall blinding success in RCTs. It yields a single value that combines blinding data from all arms and assumes that a response of ‘*Do not know*’ represents successful blinding. On the other hand, Bang’s BI is used to characterize and evaluate the blinding situation in each trial arm independently, estimating the percentage of unblinding beyond chance in each arm. An additional analysis was performed of both the initial responses and ancillary data that were collected from the subjects who initially answered ‘*Do not know*’ [25].

James’ BI ranges from 0 to 1 (0 representing total lack of blinding, 1 representing complete blinding, and 0.5 representing completely random blinding). To interpret the results, this study considered a lack of blinding if the upper bound of the confidence interval (CI) was below 0.5 [16,26]. Bang’s BI can be directly interpreted as the proportion of the unblinding in each arm. It ranges between −1 and 1, with 0 as a null value indicating the most desirable situation representing random blinding or complete blinding, 1 representing complete unblinding because all participants guess their treatment allocation correctly, and −1 representing all participants guess their treatment allocation incorrectly. Therefore, when one-sided CI did not cover the 0 value, the study was regarded as lacking blinding [25].

## 3. Results

**Figure 2:**
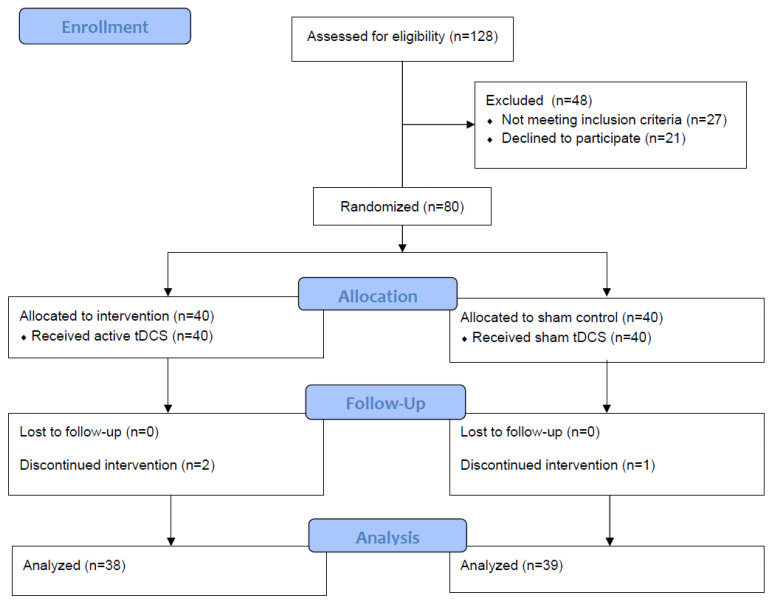
Intervention flowchart.

Seventy-seven subjects with fibromyalgia (74 women and 3 men) completed the trial, *n* = 38 in the active tDCS group and *n* = 39 in the sham stimulation group (Figure 2), for reasons unrelated to the study. No severe adverse effects were observed during the intervention. The age of the participants ranged between 34 and 65 years, with an average of 50.3 years (SD = 7.6). No differences between groups were noted in terms of demographic or clinical data at baseline (Table 1).

The guesses and ancillary data of the subjects, which were collected from those who initially answered, ‘*Do not know*’, are shown in Table 2 in a 23 table format. Table 3 shows James’ BI and Bang’s BI values obtained for the study subjects. According to the interpretation of data established by James et al. and Bang et al. [16,26], participant blinding was satisfactory when analyzed globally or as each treatment arm independently.

The guesses of the therapist and ancillary data that were additionally collected when it was initially answered ‘*Do not know*’ are shown in Table 2. Table 3 shows the values of James’ BI and Bang’s BI obtained for the therapist. According to the interpretation of data established by James et al. and Bang et al. [16,26], the blinding of the therapist was satisfactory when analyzed globally or as each study group independently.

## 4. Discussion

The results of this study reveal the successful blinding of both the participants and the therapist for the protocols used to apply active and sham tDCS to subjects with fibromyalgia. However, some differences were observed between the blinding of the subjects and the therapist applying the treatment, especially in the active tDCS group.

Blinding of subjects was found to be successful according to James’ and Bang’s BIs and similar in both the active and sham stimulation groups. Bang’s BI obtained negative values close to 0 in both study arms, with an unblinding percentage < 13%. Some trials have shown that tDCS interventions with a current intensity of 1 mA and an electrode size of 25 cm^2^ achieved reliable blinding of subjects [3,27]. Other authors have suggested that when the intensity of the applied current was > 1 mA (i.e.,: 2 mA), blinding of the subject was more difficult due to a more intense perception of the current [28,29]. However, this study followed a protocol that applied a current intensity of 2 mA with a 25 cm^2^ electrode and showed successful blinding of subjects. The fact that this protocol was applied to treatment-naive subjects can be a factor that determined the success of the blinding. Ambrus et al. observed that subjects with previous treatment experience were more likely to be able to differentiate between stimulation and non-stimulation trials and to correctly identify sham and verum stimulation conditions [26].

Although the data from both BIs suggest that blinding of the therapist was achieved, the therapist obtained a Bang’s BI lower than that obtained for the subjects in both the active and sham tDCS group. Additionally, there were differences in the blinding of the therapist between the active and sham groups: unblinding was 13% in the sham group, whereas in the active tDCS group, the therapist guessed the assignment in 21% and 20% of cases with and without ancillary data, respectively. The presence of skin erythema resulting from vasodilation caused by tDCS, mainly under the electrode placed at the supraorbital area, is one of the main factors hindering the blinding success of the therapist [29,30] and can be the reason why the therapist was capable of guessing the assignment of patients into the active tDCS group, whereas blinding was complete in the case of sham stimulation. In future research, complete blinding could be achieved provided a person outside the research team is responsible for removing the device from the subjects since the reddening disappears minutes after ending the application. Previous experience of the therapist, in contrast with treatment-naive subjects, can also explain the better blinding of the latter in the active tDCS group.

Former trials have assessed blinding using the same protocol for applying sham tDCS in patients with Alzheimer’s or cognitive disorders [31], healthy participants [28,32], or depression [33], but not in subjects with fibromyalgia. Their outcomes were contradictory depending on the pathology, finding successful blinding when assessing the protocol in Alzheimer’s or cognitive disorders [31], and inadequate blinding in healthy volunteers [28,32] or patients with depression [33]. These differences suggest that there may be factors intrinsic to the pathology, such as their cognitive condition or sensitization, that can affect the perception of the current and, therefore, the identification of the received intervention. Additionally, the statistical method for assessing the success of blinding differed among trials, with studies employing the Chi-squared test [31,34], the binomial test [34], McNemar’s tests [32], or Kappa measure of agreement [28]. To our knowledge, this is the first study that calculated James’ and Bang’s BIs for assessing how successful blinding was in an intervention with tDCS, a method that can show advantages over those employed in the above-mentioned trials. The Chi-squared, binominal, and McNemar’s tests provide *p*-values for statistical analysis but not a numerical value for quantifying blinding itself. The Cohen’s Kappa statistic coefficient measures agreement rather than disagreement, which is a more desirable outcome for blinding. Therefore, the interpretation of the Kappa coefficient was problematic [24]. Additionally, the above-mentioned trials did not take into consideration the ‘*Do not know*’ response except for that by Reckow et al. [31].

Our study has some limitations. First, the sample size was calculated based on a previous study of the effects of tDCS on pain, although the main outcome of this study is the assessment of blinding. This is because the analysis presented is secondary to a primary study analyzing the effects of tDCS on pain. Secondly, the therapist who applied the intervention was the one who removed the electrodes from the participants, being able to observe the redness of the skin if this occurred. This could be one of the reasons that would explain the lower value of the therapist’s BI.

## 5. Conclusions

In conclusion, the protocols for applying active and sham tDCS in this study have shown satisfactory blinding of the therapist and subjects with fibromyalgia who are treatment-naive for tDCS. Extrapolating the outcomes of this trial to patients with other pathologies or with previous experience in tDCS must be performed with caution. Future research employing these statistical indexes for assessing the success of blinding must be conducted in patients suffering from other pathologies where tDCS has shown to be an effective treatment. This would strengthen the current evidence and prevent biases, especially in studies where the outcome variable depends on the self-perception of subjects.

## Figures and Tables

**Table 1:** Demographic and clinical characteristics at baseline and comparison between groups.

Outcome	Active tDCS *n* = 40 Mean (SD)	Sham tDCS *n* = 40 Mean (SD)	Between-Groups Comparison *p*-Value
Age (years)	50.6 (7.0)	49.5 (8.7)	0.55
Gender (women/men)	38/2	39/1	1.0
BMI	27.2 (5.6)	26.6 (5.6)	0.59
Duration of illness (years)	11.9 (7.3)	11.3 (6.6)	0.73
Widespread pain index	13.4 (2.7)	13.6 (2.5)	0.73
Symptoms severity scale	8.7 (2.0)	8.6 (1.9)	0.77
Pain (VAS)	61.1 (14.1)	59.9 (14.4)	0.70
Disease impact (FIQ)	65.2 (14.6)	62.9 (14.8)	0.48
Anxiety (STAI-ES)	33.7 (10.2)	31.8 (11.5)	0.43
Catastrophism (PCS)	24.3 (12.3)	25.0 (11.0)	0.79
Depression (BDI-II)	18.9 (7.2)	18.9 (7.6)	1.0

BMI: Body mass index. FIQ: Fibromyalgia Impact Questionnaire. STAI-ES: State–Trait Anxiety Inventory. PCS: Pain Catastrophizing Scale. BDI-II: Beck Depression Inventory II.

**Table 2:** Guesses of the participants and the therapist, and ancillary data for ‘Do not know’ responses.

**Participant’s Guess**				
**Allocation**	**Main Data, *n* (%)**			
	Active tDCS	Sham tDCS	Do not know	Total
Active tDCS	6 (7.8)	9 (11.7)	24 (31.1)	39 (50.6)
Sham tDCS	10 (13.0)	7 (9.1)	21 (27.3)	38 (49.4)
Total	16 (20.8)	16 (20.8)	45 (58.4)	77 (100.0)
**Allocation**	**Ancillary Data, *n* (%)**			
	Active tDCS	Sham tDCS	Not answered	Total
Active tDCS	8 (17.8)	16 (35.5)	0 (0.0)	24 (53.3)
Sham tDCS	10 (22.2)	11 (24.5)	0 (0.0)	21 (46.7)
Total	18 (40.0)	27 (60.0)	0 (0.0)	45 (100.0)
**Therapist’s Guess**				
**Allocation**	**Main Data, *n* (%)**			
	Active tDCS	Sham tDCS	Do not know	Total
Active tDCS	19 (24.6)	11 (14.3)	9 (11.7)	39 (50.6)
Sham tDCS	11 (14.3)	16 (20.8)	11 (14.3)	38 (49.4)
Total	30 (39.0)	27 (35.0)	20 (26.0)	77 (100.0)
**Allocation**	**Ancillary Data, *n* (%)**			
	Active tDCS	Sham tDCS	Not answered	Total
Active tDCS	4 (20.0)	5 (25.0)	0 (0.0)	9 (45.0)
Sham tDCS	6 (30.0)	5 (25.0)	0 (0.0)	11 (55.0)
Total	10 (50.0)	10 (50.0)	0 (0.0)	20 (100.0)

**Table 3:** Results of participants and therapist blinding. Data analysis of James’ blinding index and Bang’s blinding index.

Methods	Index	*p*-Value	95% Confidence Interval	Conclusion
**Participants**				
James	0.83	1.0	0.76–0.90	Blinded
Bang—Active/2 × 3	−0.08	0.78	−0.24–0.09	Blinded
Bang—Active/2 × 3 ^a^	−0.13	0.90	−0.29–0.04	Blinded
Bang—Placebo/2 × 3	−0.08	0.77	−0.26–0.1	Blinded
Bang—Placebo/2 × 3 ^a^	−0.07	0.74	−0.26–0.11	Blinded
**Therapist**				
James	0.55	0.79	0.45–0.64	Blinded
Bang—Active/2 × 3	0.21	0.07	−0.02–0.43	Blinded
Bang—Active/2 × 3 ^a^	0.20	0.08	−0.03–0.43	Blinded
Bang—Placebo/2× 3	0.13	0.17	−0.09–0.35	Blinded
Bang—Placebo/2 × 3 ^a^	0.13	0.18	−0.10–0.35	Blinded

^a^ Showed results with incorporation of ancillary data for ‘Do not know’ responses.

## Data Availability

The dataset generated and/or analyzed during the current study is available in the Zenodo repository (web link: 4fad18e5fd3f9a52f5927d0de809cfee).
